# Robot-assisted laparoscopic Anderson–Hynes pyeloplasty for ureteropelvic junction obstruction

**DOI:** 10.1007/s11701-024-02098-z

**Published:** 2024-09-28

**Authors:** Ann Kortbæk Bersang, Badal Sheikho Rashu, Malene Hartwig Niebuhr, Mikkel Fode, Frederik Ferløv Thomsen

**Affiliations:** Department of Urology, Copenhagen University Hospital, Herlev and Gentofte Hospital, Herlev, Denmark

**Keywords:** Ureteropelvic junction obstruction, Pyeloplasty, Anderson–Hynes, Robot assisted

## Abstract

**Objectives:**

To explore surgical, functional, and symptomatic outcomes in a series of patients who underwent robot-assisted laparoscopic Anderson–Hynes pyeloplasty (RALP) for ureteropelvic junction obstruction using the DaVinci Si surgical robotic system.

**Methods:**

Retrospective study including patients aged 16 years or older who underwent RALP from June 2016 to December2021. The following outcomes were recorded: operative outcome and complications [classified according to the Clavien–Dindo Classification (CD)] within 30 days of the procedure as well as 1 year success rate and restenosis during follow-up.

**Results:**

In total, 194 patients were available for analyses with a median follow-up of 4.5 (IQR 3.0-6.0) years. The primary indications were loss of kidney function (45%), pain (36%), infection (11%), kidney stone (6%), and others (2%). The median operation time was 134 min (IQR 112-159), the median length of stay was 2 days (IQR 2-2), and the median time with double-j stent postoperatively was 24 days (IQR 22-27). Overall, 65 out of 194 patients (33%) experienced a postoperative complication (12% CD I, 13% CD II, 8% CD IIIa or IIIb). The 1 year success rate was 92% for patients treated because of deteriorating renal function, 78% for patients treated because of symptoms, 82% for patients treated because of infections, and 78% for patients treated because of kidney stones. Seven percent of the patients presented a recurrent ureteropelvic junction stricture during follow-up.

**Conclusions:**

In our experience, robot-assisted laparoscopic Anderson–Hynes pyeloplasty performed with the DaVinci Si system is a safe with a few major complications and acceptable success rate.

## Introduction

Ureteropelvic junction obstruction (UPJO) is a common urologic condition resulting in impeded urine flow from the pelvis to the proximal ureter and progressive dilation of the renal collecting system. The obstruction can be either of congenital or acquired origin [1]. The typical clinical presentation of an UPJO is renal colic and back pain, which can be exacerbated by increased fluid intake or infections [1, 2]. Indications for surgical treatment are recurrent flank pain, kidney stone formation, recurrent infections, and loss of renal function [3].

The Anderson–Hynes technique was first described in 1949 [4] and the first laparoscopic pyeloplasty was described in 1993 [5]. The advantages of the laparoscopic technique are fewer postoperative complications, less pain, and shorter hospitalizations comparable to open pyeloplasty [6]. In 2002, the first robot-assisted laparoscopic Anderson–Hynes pyeloplasty (RALP) was described [7].

Postoperative [8–13] and renal function outcomes [8–16] have been reported in several publications, but the effect of RALP in patients treated because of symptoms, infections, or stone formation is sparse [17]. The objective of this study was to evaluate the safety of RALP for UPJO and report outcomes 1 year after the procedure.

## Material and methods

We performed a single-center retrospective cohort study where we included patients aged 16 years or older who underwent RALP performed on the DaVinci Si system (Intuitive Surgical, Inc.) from June 2016–December 2021.

The procedure was performed with incision of the renal pelvis and subsequent spatulation of the ureter along the lateral surface approximately 5 mm beyond the obstructed part. In cases with crossing vessels, the ureter was moved anteriorly to the vessels. The anastomosis was performed with vicryl 4-0 or monocryl violet 4-0 sutures. After completing the posterior wall of the ureter, a double-J ureteral stent was inserted antegraded though the trocar using a guidewire and its placement in the bladder was confirmed with reflux of blue dyed water [18]. The double-J ureteral stent was only changed in patients who had a stent prior to RALP in case of incrustations, if it had been in place for more than 4 months if the surgeon deemed it necessary. The double-J stent was routinely removed 4 weeks after RALP in the out-patient clinic using a flexible cystoscope.

In case of kidney stone, extraction was performed through a laparoscopic port using a flexible ureteroscope. All patients received one dose of prophylactic IV antibiotic perioperative.

Patients were identified from electronic records by the surgical code for RALP. The following data were recorded following electronic patient chart review: demographic information including age, gender, smoking status, BMI, ASA score, and Charlson comorbidity index (CCI); preoperative information including flank pain, infection, recurrent stone formation, preoperative decompression with double-J stent or nephrostomy, CT scan findings (presentation of crossing vessel) and results of last MAG3 renal scan before RALP. We have exclusively noted the primary indication for treatment, despite some patients naturally presenting with more than one problem relating to the UPJO. In case of preoperative decompression, this was kept until definitive RALP. Operative details included: operations time, crossing vessels, and perioperative complications. Postoperative details included: length of stay, time with double-J ureteral stent, and complications within 30 days of surgery.

Complications were classified according to the Clavien–Dindo classification (CD) [19]. Postoperative success was evaluated 1 year after the procedure. Postoperative renal function was assessed with MAG3 renal scan and recorded as improved, stable or deteriorated. Symptoms, defined as pain, were recorded as improved, unchanged or aggravated. Patients treated because of stones underwent CT scan following 1 year. Surgeries were defined as successful if patients treated because of deteriorating renal function had stable or improved renal function, if symptoms had improved in patients treated for pain, while for patients with stones or infection, success was defined as being stone- or infection-free with stable or improved kidney function at 1-year follow-up.

Finally, recurrent UPJOs and their treatments were also recorded.

The study received ethical and legal approval from the regional center for register research of the Capital Region of Denmark according to Danish law (journal number: R-22013482). The manuscript was prepared according to the STROBE statement (www.strobe-statement.org).

### Statistics

Descriptive statistics were used. Median follow-up was calculated with the reverse Kaplan–Meier method [20]. Statistical analysis was performed with Excel and R version 4.2.1 (R Foundation for Statistical Computing, Vienna, Austria).

## Results

Overall, 208 patients were identified based on the surgical code for RALP. Of these, 194 patients were included in the analysis of operative outcome and postoperative complications, while 182 patients were available for 1 year postsurgical evaluation. The following 14 patients were excluded: 2 due to different pyeloplasty techniques being used (YV: *n* = 1, a.m. Heineke–Mikulicz: *n* = 1), 8 who underwent a re-do pyeloplasty, and 4 with no postsurgical follow-up data at all.

The median age at the time of surgery was 38 years (IQR 27–58) and 111 (57%) were female. The median BMI was 23 (IQR 21–26), the median ASA score was 1 (IQR 1–2) and the median CCI was 0 (IQR 0–2), Table 1. The median follow-up time following surgery was 4.5 (IQR 3.0–6.0) years.

**Table 1:** Baseline characteristics of 194 patients undergoing RALP for UPJO

	*n*** (%)**
Gender	
Female	111 (57%)
Male	83 (43%)
Charlson comorbidity index	
0	115 (59%)
1	27 (14%)
2 +	52 (27%)
ASA classification	
1	113 (58%)
2	67 (34%)
3 +	14 (7%)
Body mass index	
< 25	102 (52%)
25–29	46 (24%)
30 +	46 (24%)
Smoking	
Yes	37 (19%)
No	157 (81%)
Preoperative decompression	
None	70 (36%)
Nephrostomy	19 (10%)
Stent	105 (54%)

The primary indication for RALP was deteriorating renal function in 88/194 patients (45%), of which 61/88 patients (69%) had a double-J ureteral stent inserted prior to RALP to protect the renal function. Flank pain was the indication for 70/194 (36%), of which 27/70 patients (38%) had a double-J stent prior to RALP to decrease pain. Infection was the indication for 22/194 (11%) patients, of which 17 patients required a nephrostomy or double-J stent as a treatment for pyonephrosis prior to RALP. In 11/194 patients (6%), recurrent formation of kidney stones was the indication. Earlier stone surgery induced a UPJO resulting in flank pain for two patients and one patient was evaluated because of flank pain and diagnosed with a UPJO following ureteroscopy under the suspicion for upper urothelial cancer.

The median operation time was 134 min (IQR 112–159) and 119/194 (61%) had crossing vessels. The median length of stay was 2 days (IQR 2–2) and the median time with double-J stent postoperatively was 24 days (IQR 22–27).

No major perioperative complications occurred and none of the procedures were converted to open surgery. Twenty-four of one hundred ninety-four patients (12%) experienced a CD I complication, twenty-six patients (13%) experienced a CD II, and fifteen patients (8%) experienced a CD grade IIIa or IIIb complication, Table 2. Of note, none of the patients who received IV antibiotics for postoperative fever had positive blood culture. Two patients developed abscesses after 5 and 7 days, respectively, and required percutaneous drainage. Five patients required nephrostomy to treat uroplania. In six patients, the double-J ureteral stent was displaced and removed with a ureteroscope.

**Table 2:** Overview of complications classified according to the Clavien–Dindo score

	Clavien–Dindo score					
Complication	I	II	IIIa	IIIb	IV	V
	*n* (%)	*n* (%)	*n* (%)	*n* (%)	*n* (%)	*n* (%)
Pain, worse than expected	18					
Nauseas	3					
Catheter	2					
Low blood pressure (IV fluid)	1					
Febrile (IV antibiotic)		11				
Urinary tract infection (PO antibiotic)		8				
Low hemoglobin without active bleeding		3				
Dyspnea		1				
Blurry vision		1				
Ventricle retention (probe)		1				
Wound infection		1				
Uroplania (nephrostomy)			5			
Abscess (percutaneous drainage)			2			
Suturing of port defect			1			
Displaced stent (ureteroscopy)				6		
Incrustations on stent (ureteroscopy)				1		
					0	0
In total	24 (12%)	26 (13%)	8 (4%)	7 (3%)	0	0

The renal function following 1 year could be assessed in 85 of the 88 patients who were treated because of deteriorating renal function. In total, 78/85 (92%) had stable or improved function following the procedure, while 8% lost additional renal function. Effect on symptoms following 1 year could be assessed in 63 of the 70 patients who were treated because of flank pain. In total, 78% (49/63) had improvement of their symptoms, while symptoms were unchanged in 21% (13/63) and 1% (1/63) had aggravation. Eighteen of twenty-two (82%) patients treated because of infection had not had a recurrent infection at 1-year follow-up. One patient experienced pyonephrosis within 30 days, but also had stone formation that was not removed during RAPL. After retrograde intrarenal stone removal, the patient did not experience any additional infections. Three patients still suffered from lower urinary tract infections but did not experience pyelonephritis. Seven of nine patients (78%) were stone free at the 1-year follow-up—two patients were lost to follow-up. In one patient, stone extraction was not possible due to narrow calyces. Another patient had an unchanged number of small kidney stones at follow-up.

In total, 14/194 (7%) patients had a recurrent UPJO during follow-up, of which 4 patients had a new intervention: 2 patients underwent re-do RALP, 2 patients underwent endopyelotomy, with one of these failing endopyelotomy and requiring a subsequent RALP. In total, seven patients lost all renal function on the affected kidney. Two of these underwent a subsequent nephrectomy because of pain.

## Discussion

In this retrospective study of RAPL, we found 92% success rate for patients treated because of deteriorating renal function. Comparable numbers were: 78% for patients treated because of pain—with 1% experiencing aggravation of their symptoms—82% for patients treated because of infections, and 78% for patients treated because of kidney stone. The procedure was safe with few major (CD III) and no serious (CD IV-V) complications.

The main limitation of this study is the retrospective design. Renal outcomes, which were measured objectively with MAG3 renal scan, are more likely to be generalizable than the symptomatic outcomes which was measured by retrospective patient chart review and could be affected by re-call biases [21].

Treatment of UPJO has rapidly developed over the last 2 decades from open pyeloplasty to minimal invasive surgery [6, 7]. Laparoscopic pyeloplasty and RALP are associated with reduced pain, fewer postoperative complications, and shorter hospital stay compared to an open procedure [22, 23]. Other advantages of RALP are three-dimensional vision, tremor filtering, motion scaling, and articulating movements with greater degrees of freedom [23–25]. Because of these advantages, RALP has shown a faster learning curve, with fewer cases needed to achieve a plateau in operation time compared to laparoscopic pyeloplasty [26].

The observed CD I and II complications are higher than previous studies [9, 27], whereas the 7% major complications are in line with the 3–11% major complications in previous reports [9, 12, 17]. Minor complications such as fever and urinary tract infections are manageable and expected. Moreover, we registered postoperative pain as a CD I complication where other studies potentially have not included this as a complication [9, 28].

Following 1 year, 92% of patients treated because of deteriorating renal function had improved or stable function following the procedure, while 8% lost additional renal function. Previous studies report 69–100% renal success rates with the majority between 90–95% [8–16, 18, 29]. In patients treated because of pain, 78% had fewer symptoms, which is lower than the only previous study reporting on symptom outcomes [17]. This study found a 94% improvement of symptoms in a cohort of 88 patients but did not specify when symptoms were recorded postoperatively [17].

We used the last renal scintigraphy before pyeloplasty in patients who had a double-J ureteral stent prior to the procedure because we wanted to compare the renal function following 1 year with the status at the time of the procedure to be able to fairly counsel our patients as to what they can expect from undergoing the procedure. Thus, it would be interesting to investigate whether a preoperative double-J stent and duration of the decompression affect the success rate. Unfortunately, our cohort is too small to perform any reliable analysis on this topic.

We found 7% had a recurrent UPJO during follow-up, which previously has been reported between 2–4% [9, 12]. Finally, in line with previous reports, 3.6% of the patients ended up with a solitary functioning kidney [9, 13].

## Conclusion

Robot-assisted laparoscopic Anderson–Hynes pyeloplasty demonstrates an acceptable safety and a high renal success rate and moderate symptomatic success rate in treating UPJO with few patients having deteriorated renal function or aggravation of symptoms. However, we were unable to find the same impressive improvement in pain-related symptoms as a previous study and more than 1/20 experienced a recurrence of UPJO. Thus, the surgical treatment of UPJO warrants further investigation preferably in prospective design with validated objective symptom score.

## Data Availability

No datasets were generated or analyzed during the current study.

## References

[1] Paraboschi I, Mantica G, Dalton NR, Turner C, Garriboli M (2020) Urinary biomarkers in pelvic-ureteric junction obstruction: A systematic review. Transl Androl Urol 9(2):722–742. 10.21037/tau.2020.01.01 32420179 10.21037/tau.2020.01.01 PMC7215034

[2] Khan F, Ahmed K, Lee N, Challacombe B, Khan MS, Dasgupta P (2014) Management of ureteropelvic junction obstruction in adults. Nat Rev Urol 11(11):629–638. 10.1038/nrurol.2014.240 25287785 10.1038/nrurol.2014.240

[3] Kausik S, Segura JW (2003) Surgical management of ureteropelvic junction obstruction in adults. Int Braz J Urol 29(1):3–10. 10.1590/S1677-55382003000100002 15745460 10.1590/s1677-55382003000100002

[4] Anderson JC, Hynes W (1949) RETROCAVAL URETER: a case diagnosed pre-operatively and treated successfully by a plastic operation. Br J Urol 21(3):209–214. 10.1111/j.1464-410X.1949.tb10773.x 18148283 10.1111/j.1464-410x.1949.tb10773.x

[5] Schuessler WW, Grune MT, Tecuanhuey LV, Preminger GM (1993) Laparoscopic dismembered pyeloplasty. J Urol: Off J Am Urol Assoc 150(6):1795–1799. 10.1016/s0022-5347(17)35898-6 10.1016/s0022-5347(17)35898-6 8230507

[6] Moore RG, Bauer JJ, Bishoff JT (1999) LAPAROSCOPIC VERSUS OPEN PYELOPLASTY ASSESSMENT OF OBJECTIVE AND SUBJECTIVE OUTCOME. J Urol 162(3–I):692–695 10458344 10.1097/00005392-199909010-00016

[7] Gettman MT, Peschel R, Neururer R, Bartsch G, Rassweiler J (2002) A comparison of laparoscopic pyeloplasty performed with the davinci robotic system versus standard laparoscopic techniques: Initial clinical results. Eur Urol 42(5):453–458. 10.1016/S0302-2838(02)00373-1 12429153 10.1016/s0302-2838(02)00373-1

[8] Moreno-Sierra J, Castillon-Vela I, Ortiz-Oshiro E et al (2013) Robotic Anderson-Hynes dismembered pyeloplasty: initial experience. Int J Med Robot + Comput Assist Surg 9(2):127–133. 10.1002/rcs.1473 10.1002/rcs.1473 23408585

[9] Masieri L, Sforza S, Mari A et al (2019) Robot-assisted pyeloplasty for ureteropelvic junction obstruction: Experience from a tertiary referral center. Minerva Urol Nefrol 71(2):168–173. 10.23736/S0393-2249.19.03328-9 30767492 10.23736/S0393-2249.19.03328-9

[10] Danuser H, Germann C, Pelzer N, Rühle A, Stucki P, Mattei A (2014) One- vs 4-week stent placement after laparoscopic and robot-assisted pyeloplasty: results of a prospective randomised single-centre study. BJU Int 113(6):931–935. 10.1111/bju.12652 24472002 10.1111/bju.12652

[11] Iwamura M, Nishi M, Soh S et al (2013) Efficacy and late complications of laparoscopic pyeloplasty: experience involving 125 consecutive ureters. Asian J Endosc Surg 6(2):116–121. 10.1111/ases.12007 23206265 10.1111/ases.12007

[12] Castillo OA, Cabrera W, Aleman E, Vidal-Mora I, Yañez R (2014) Laparoscopic pyeloplasty: technique and results in 80 consecutive patients. Actas Urológicas Españolas (English Edition) 38(2):103–108. 10.1016/j.acuroe.2013.11.003 10.1016/j.acuro.2013.04.010 23910728

[13] Gargouri MM, Nouira Y, Kallel Y et al (2013) The long-term results of laparoscopic retroperitoneal pyeloplasty in adults. Arab J Urol 11(4):411–414. 10.1016/j.aju.2013.09.001 26558113 10.1016/j.aju.2013.09.001 PMC4442994

[14] Elbaset MA, Osman Y, Elgamal M et al (2021) Long-term outcomes after pyeloplasty for pelvi-ureteric junction obstruction in adults associated with renal congenital anomalies: Age, sex and renal function matched analysis. Arab J Urol 19(2):173–178. 10.1080/2090598X.2020.1816600 10.1080/2090598X.2020.1816600 PMC8158229 34104493

[15] Kumar S, Bhirud DP, Mittal A, Navriya SC, Ranjan SK, Mammen KJ (2021) Robot-assisted laparoscopic pyeloplasty: A retrospective case series review. J Minim Access Surg 17(2):202–207. 10.4103/jmas.JMAS_10_20 32964889 10.4103/jmas.JMAS_10_20 PMC8083735

[16] Seo IY, Oh TH, Lee JW (2014) Long-term follow-up results of laparoscopic pyeloplasty. Korean J Urol 55(10):656–659. 10.4111/kju.2014.55.10.656 25324948 10.4111/kju.2014.55.10.656 PMC4198764

[17] Erdeljan P, Caumartin Y, Warren J et al (2010) Robot-assisted pyeloplasty: Follow-up of first Canadian experience with comparison of outcomes between experienced and trainee surgeons. J Endourol 24(9):1447–1450. 10.1089/end.2009.0617 20677915 10.1089/end.2009.0617

[18] Moretto S, Gandi C, Bientinesi R et al (2023) Robotic versus open pyeloplasty: perioperative and functional outcomes. J Clin Med. 10.3390/jcm12072538 37048622 10.3390/jcm12072538 PMC10095392

[19] Dindo D, Demartines N, Clavien PA (2004) Classification of surgical complications: A new proposal with evaluation in a cohort of 6336 patients and results of a survey. Ann Surg 240(2):205–213. 10.1097/01.sla.0000133083.54934.ae 15273542 10.1097/01.sla.0000133083.54934.ae PMC1360123

[20] Schemper M, Smith TL (1996) A note on quantifying follow-up in studies of failure time. Control Clin Trials 17(4):343–346 8889347 10.1016/0197-2456(96)00075-x

[21] Talari K, Goyal M (2020) Retrospective studies—utility and caveats. J R Coll Physicians Edinb 50(4):398–402. 10.4997/jrcpe.2020.409 33469615 10.4997/JRCPE.2020.409

[22] Fiori C, Bertolo R, Manfredi M et al (2017) Robot-assisted laparoendoscopic single-site versus mini-laparoscopic pyeloplasty: A comparison of perioperative, functional and cosmetic results. Minerva Urol Nefrol 69(6):604–612. 10.23736/S0393-2249.17.02833-8 28429925 10.23736/S0393-2249.17.02833-8

[23] Eden CG (2007) Minimally invasive treatment of ureteropelvic junction obstruction: a critical analysis of results. Eur Urol 52(4):983–989. 10.1016/j.eururo.2007.06.047 17629395 10.1016/j.eururo.2007.06.047

[24] Eichel L, Ahlering TE, Clayman RV (2004) Role of robotics in laparoscopic urologic surgery. Urologic Clin N. Am 31(4 SPEC. ISS.):781–792. 10.1016/j.ucl.2004.06.014 10.1016/j.ucl.2004.06.014 15474606

[25] Mantica G, Ambrosini F, Parodi S, Tappero S, Terrone C (2020) Comparison of safety, efficacy and outcomes of robot assisted laparoscopic pyeloplasty vs conventional laparoscopy. Res Rep Urol 12:555–562. 10.2147/RRU.S238823 33204662 10.2147/RRU.S238823 PMC7667144

[26] Andolfi C, Lombardo AM, Aizen J et al (2022) Laparoscopic and robotic pyeloplasty as minimally invasive alternatives to the open approach for the treatment of uretero-pelvic junction obstruction in infants: a multi-institutional comparison of outcomes and learning curves. World J Urol 40(4):1049–1056. 10.1007/s00345-022-03929-0 35044490 10.1007/s00345-022-03929-0

[27] Palese MA, Stifelman MD, Munver R et al (2005) Robot-assisted laparoscopic dismembered pyeloplasty: A combined experience. J Endourol 19(3):382–386. 10.1089/end.2005.19.382 15865532 10.1089/end.2005.19.382

[28] Mufarrij PW, Shah OD, Berger AD, Stifelman MD (2007) Robotic reconstruction of the upper urinary tract. J Urol 178(5):2002–2005. 10.1016/j.juro.2007.07.018 17869303 10.1016/j.juro.2007.07.018

[29] Whiting D, Whitehurst L, Tsang D, Hussain M, Barber N, Malki M (2021) MP53–05 RETROPERITONEAL ROBOTIC-ASSISTED LAPAROSCOPIC PYELOPLASTY: A 10 YEAR EXPERIENCE IN A SINGLE INSTITUTION. J Urol. 10.1097/JU.0000000000002083.05 34931543 10.1089/end.2021.0551

